# Supplementary material for: Cholinergic signaling modulates intestinal pathophysiology in a Drosophila model of cystic fibrosis
Source: PLoS Genet. 2026 Feb 17;22(2):e1012048. doi: 10.1371/journal.pgen.1012048 (PMC12923121; doi:10.1371/journal.pgen.1012048)
Supplement: S1 Table — (PDF) [file pgen.1012048.s006.pdf]

TF2TG							
**TF FBgn**	**TF Symbol**	**Peak Count**	**Motif Count**	**REDfly TFBS Count**	**Location**	**Protein-Protein Interactors**	**Genetic Interactors**
FBgn0000659	fkh	1	2	0	intragenic	Ada2b	
FBgn0000659	fkh	1	2	0	upstream	Ada2b	
FBgn0035625	Blimp-1	2	5	0	upstream		
FBgn0035625	Blimp-1	0	7	0	intragenic		
FBgn0011236	ken	0	1	0	intragenic	pzg, Ada2b, E(bx), Trl	
FBgn0011236	ken	0	1	0	upstream	pzg, Ada2b, E(bx), Trl	
FBgn0000286	Cf2	0	1	0	intragenic	bin	
FBgn0027339	jim	0	20	0	intragenic		
FBgn0027339	jim	0	1	0	upstream		
FBgn0283451	br	1	0	0	upstream	Ada2b, Rel, rib	Met (pubmed)
FBgn0283451	br	0	1	0	intragenic	Ada2b, Rel, rib	Met (pubmed)
FBgn0264490	Eip93F	0	1	0	intragenic		
FBgn0261113	Xrp1	1	0	0	upstream	Irbp18	
FBgn0261113	Xrp1	0	2	0	intragenic	Irbp18	
FBgn0085424	nub	0	5	0	intragenic	pdm2, wek	
FBgn0085424	nub	0	4	0	upstream	pdm2, wek	
FBgn0032223	GATAd	0	5	0	upstream	mam, Ada2b	
FBgn0262582	cic	2	0	0	upstream	gro	Dl (pubmed)
FBgn0262582	cic	0	1	0	intragenic	gro	Dl (pubmed)
FBgn0005660	Ets21C	0	1	0	upstream		
FBgn0014018	Rel	2	1	0	upstream	htk, Dif, dl, pzg, br, grh, sqz	
FBgn0004394	pdm2	0	3	0	intragenic	salr, nub	
FBgn0004394	pdm2	0	4	0	upstream	salr, nub	
FBgn0032587	CG5953	0	1	0	upstream	knrl	
FBgn0263108	BtbVII	0	1	0	intragenic	CG32121	
FBgn0013263	Trl	3	0	0	intragenic	CG12155, CG8924, psq, Ssrp, ken, pzg, E2f1, bab2, lola, E(bx), Gug, Adf1, ttk	
FBgn0013263	Trl	2	0	0	upstream	CG12155, CG8924, psq, Ssrp, ken, pzg, E2f1, bab2, lola, E(bx), Gug, Adf1, ttk	
FBgn0264490	Eip93F	0	1	0	intragenic		

Supplementary Table 1: Related to Figure 5

TF2TG results for TFs with ChIP-seq Peak counts and/or binding motifs within 5 kb of Ace gene that were included in the transcription factor screen for Ace transcription (Fig 5A) .
